# Global analysis of lysine 2-hydroxyisobutyrylation during *Fusarium graminearum* infection in maize

**DOI:** 10.3389/fpls.2022.1000039

**Published:** 2022-09-15

**Authors:** Kang Zhang, Hongzhe Cao, Yuxin Ma, Helong Si, Jinping Zang, Hua Bai, Lu Yu, Xi Pang, Fan Zhou, Jihong Xing, Jingao Dong

**Affiliations:** ^1^State Key Laboratory of North China Crop Improvement and Regulation, Hebei Agricultural University, Baoding, Hebei, China; ^2^Hebei Key Laboratory of Plant Physiology and Molecular Pathology, Hebei Agricultural University, Baoding, Hebei, China

**Keywords:** maize, proteomic, lysine 2-hydroxyisobutyrylation, histone modification, disease resistance

## Abstract

Proteins post-translational modification (PTMs) is necessary in the whole life process of organisms. Among them, lysine 2-hydroxyisobutyrylation (Khib) plays an important role in protein synthesis, transcriptional regulation, and cell metabolism. Khib is a newly identified PTM in several plant species. However, the function of Khib in maize was unclear. In this study, western blotting results showed that Khib modification level increased significantly after *Fusarium graminearum* infection, and 2,066 Khib modified sites on 728 proteins were identified in maize, among which 24 Khib sites occurred on core histones. Subcellular localization results showed that these Khib modified proteins were localized in cytoplasm, chloroplast, and nucleus. Then, comparative proteomic analysis of the defense response to *F. graminearum* infection showed that Khib modification participated in plant resistance to pathogen infection by regulating glycolysis, TCA cycle, protein synthesis, peroxisome, and secondary metabolic processes, such as benzoxazinoid biosynthesis, phenylpropanoid biosynthesis, jasmonic acid synthesis, and tyrosine and tryptophan biosynthesis. In addition, we also demonstrated that lysine 2-hydroxyisobutyrylation sites on histones were involved in the gene expression of pathogenesis-related proteins. Our results provide a new perspective for the study of plant disease resistance, and had directive significance of maize disease resistance for molecular breeding.

## Introduction

Proteins post-translational modification (PTM) can change the charge, conformation and molecular weight of proteins by adding chemical groups to the amino acid residues of proteins (Lin and Caroll, 2018), which play important roles in transcriptional regulation, metabolic regulation, protein interaction, and many other biological processes (Choudhary et al., 2014; Muller, 2018; Stocks and Zierath, 2021). With the continuous understanding of the significance of PTMs, more and more post-translational modifications of lysine have been found, such as lysine succinylation, malonylation, crotonylation, β-hydroxybutyrylation, glutarylation and lactylation (Chen et al., 2007; Xie et al., 2012, 2016; Xu et al., 2018; Zhao et al., 2018). The 2-hydroxyisobutyrylation of lysine (Khib) was first reported as a widely distributed active histone mark (Dai et al., 2014). Later, 2-hydroxyisobutyrylation of lysine was found not only in HeLa cells, but also in mouse, *Drosophila* and *Saccharomyces cerevisiae* (Dai et al., 2014; Huang et al., 2017, 2018). With the rapid development of separation methods and mass spectrometry technology, liquid chromatography-mass spectrometry (HPLC-MS/MS) has become an important means to study proteomics and protein post-translational modification (Van Gool et al., 2020; Virág et al., 2020). Due to its high sensitivity, high throughput and high efficiency, mass spectrometry can analyze post-translational modification groups, interpret modification sites, quantify modification abundance, and analyze modification-mediated protein complexes (Chicooree et al., 2015; Liu et al., 2019). Therefore, mass spectrometry-based proteomics can systematically identify the distribution characteristics of Khib in organisms, reveal its biological functions, and discover regulatory enzymes and their regulatory mechanisms (Dai et al., 2014; Cheng et al., 2020). Global profiling of the Khib proteome in mammalian cells is first reported and 6,548 Khib sites on 1,725 proteins are identified (Huang et al., 2018). Although some progress has been made in the research on Khib, it has been mainly in mammalian cells and yeast, while studies in plants are still limited. Therefore, it is interesting to determine the function of Khib proteins in plants. In rice, 9,916 Khib modification sites are identified on 2,512 proteins in seeds, while 4,163 Khib sites are identified on 1,596 proteins of rice seedlings, which were involved in a variety of biological functions and metabolic processes (Meng et al., 2017; Xue et al., 2020). In wheat, 1,074 proteins are identified by proteomics technology, including 3,348 Khib lysine modification sites, and the key regulation of Khib modification of K206 on PGK on enzyme activity is proved (Bo et al., 2021; Zhang et al., 2021). Total of 1,525 proteins with 4,333 Khib-modified peptides are identified in Chinese herb rhubarb (Qi et al., 2021). Recent study analyzes the overall changes of protein abundance and Khib modification level after pathogen infection in rice, and shows that the Khib modification level on protein after *Ustilaginoidea virens* infection is significantly reduced (Chen et al., 2021). However, the correlation between histones and non-histone proteins with Khib modification in the process of maize resistance to pathogen infection need to be supplemented, and whether proteins are selectively modified by 2-hydroxyisobutyrylation in maize disease resistance remains to be explored.

Maize is an important food and economic crops and susceptible to a variety of pathogens during growth and development (Xu et al., 2012; Sanchez et al., 2014). However, very little is known about protein post-translational modification on histones and non-histone proteins in maize and the responses against *Fusarium graminearum* infection. In this study, we used high-throughput TMT-based technology for proteomics comparison to explore protein lysine 2-hydroxyisobutyrylation modification and their biological processes in maize stems, which showed moderate resistance to *F. gramincarum* infection. Totally, 2,066 Khib-modified peptides on 728 proteins were identified in our proteomics data. Functional analysis results showed that proteins with different Khib modification were involved in many important biological processes. In addition, we found that Khib modification on PR proteins was involved in disease resistance in maize. These results provided a new understand to Khib modification and explores the function of the identified proteins in plant disease resistance.

## Materials and methods

### Plant materials and treatments

The maize inbred line B73 line was grown in the greenhouse in Hebei Agricultural University (Baoding, Hebei province, China). *Fusarium graminearum* was grown on PDA plates at 28°C for 6 days. Conidial suspensions were harvested by adding sterile distilled water containing 0.05% (v/v) Triton X-100 and scraping the plates using a glass spreader. The concentration of *F. gramincarum* conidia was quantified using a hemocytometer and diluted to 1 × 10^6^ spores/ml for inoculation. A micropipette was used to inoculate the second or third internodes of maize stems above the soil surface, and then 30 μl conidial suspension was injected. Wounds are covered with sterile gauze to retain moisture and avoid contamination by other organisms. The histone deacetylase inhibitor Trichostatin A (TSA, MedChemExpres) was treated at a concentration of 15 μmol/L, and then spraying on maize leaves at V6 stage. After treatment for 2 days, samples were collected into liquid nitrogen and placed in −80°C refrigerator for western blotting.

### Protein extraction, quantification and digestion

The sample was grinded into powder by liquid nitrogen and then transferred to 10 ml centrifuge tube. Then, lysis buffer (including 1% Protease Inhibitor Cocktail, 10 mM dithiothreitol, 3 μM TSA, 50 μM PR-619, 1% TritonX-100, 2 mM EDTA, and 50 mM NAM) was added to the powder, followed by sonication three times on ice using a high intensity ultrasonic processor (Scientz). After an equal volume of Tris-saturated phenol (pH 8.0) was added, the mixture was further vortexed for 5 min. After centrifugation (4°C, 10 min, 5,000*g*), the upper phenol phase was transferred to a new centrifuge tube. Proteins were precipitated by adding at least four volumes of ammonium sulfate-saturated methanol and incubated at −20°C for at least 6 h. After centrifugation at 4°C for 10 min, the supernatant was discarded. The remaining precipitate was washed with ice-cold methanol once, followed by ice-cold acetone for three times. The protein was resuspended in 8 M urea (Sigma), and then the redissolved protein concentration was detected with BCA kit according to the manufacturer’s instructions. Protein solution was digested with 5 mM dithiothreitol (Sigma) for 30 min at 56°C and alkylated with 11 mM iodoacetamide (Sigma) for 15 min in darkness at room temperature. Then, the protein samples were diluted by adding 100 mM TEAB to lower than 2 M urea. Trypsin (Promega) was added at the mass ratio of trypsin to protein of 1:50 for the first digestion overnight, and the mass ratio of trypsin to protein of 1:100 was added for the second digestion for 4 h.

### TMT labeling and peptide fractionation

After trypsin digestion, the trypsin-hydrolyzed peptides were desalted by Strata X C18 SPE columns (Phenomenex) and vacuum freeze-dried. The peptides were dissolved by 0.5 M TEAB and labeled according to TMT kit (Thermo Fisher Scientific) according to the manufacturer’s introduction. The labeled reagent was thawed and dissolved in acetonitrile. After mixing with the peptide, it was incubated at room temperature for 2 h. After mixing with the labeled peptide, it was desalted and vacuum freeze-dried. The tryptic peptides were fractionated into fractions by high pH reverse-phase HPLC using Agilent 300 Extend C18 column (5 μm particles, 4.6 mm ID, and 250 mm length). Then, the peptides were combined into 18 components with a gradient of 8%–32% acetonitrile (pH 9.0) and dried by vacuum centrifugation.

### Khib modification enrichment

The peptides were dissolved in IP buffer solution (100 mM NaCl, 1 mM EDTA, 50 mM Tris-HCl, 0.5% NP-40, pH 8.0). The supernatant was transferred to the washed 2-hydroxyisobutyryl resin (PTM Biolabs, PTM-904) and placed on a rotary shaker at 4°C, gently shaking and overnight incubation. After incubation, the resin was washed four times with IP buffer solution and washed twice with deionized water. Finally, 0.1% trifluoroacetic acid eluent was used to elute the resin-binding peptide, and a total of three elutions were performed. The eluent was collected and dried by vacuum freezing. After drying, desalted according to the C18 ZipTips (Millipore) instructions, vacuum freeze-dried for liquid chromatography-mass spectrometry analysis.

### LC–MS–MS analysis

Trypsin peptides are dissolved in 0.1% formic acid and directly loaded on a self-made reversed-phase analytical column (15 cm length, 75 μm i.d.). The gradient of solvent B (0.1% formic acid in 98% acetonitrile) was gradually increased from 6% to 23% through 26 min, increased from 23% to 35% over 8 min, climbing to 80% in 3 min and then maintained at 80% for 3 min, all at a constant flow rate of 400 nl/min on an EASY-nLC 1200 HPLC systems. The peptides were subjected to NSI source followed by tandem mass spectrometry (MS/MS) in Q Exactive™ Plus (Thermo) coupled online to the UPLC. 2.0 kV electrospray voltage was applied. The intact peptides were detected in the Orbitrap, with 350–1,600 m/z full scan range at 50,000 resolution. By using NCE setting as 28, peptides were selected for MS/MS, and the fragments were detected in the Orbitrap at a resolution 15,000. The data correlation process alternates between one MS scan and the subsequent 20 MS/MS scans with an interval of 15.0 s. Automatic gain control was set at 5E4. Fixed first mass was set as 100 m/z.

### Database search and bioinformatics analysis

Maxquant (v.1.5.2.8) software were used to process the MS/MS data. Tandem mass spectra were searched against MaizeGDB (V4) database concatenated with reverse decoy database. Trypsin/P is designated as a cleavage enzyme allowing up to two deletions. The mass tolerance of the parent ion is set to 20 ppm in the first search, 5 ppm in the main search and 0.02 Da in the fragment ion. The aminoformylmethyl on Cys was designated as fixed modification, and the oxidation on Met was designated as variable modification. The lowest fraction of peptide was set as >40, and the FDR was adjusted to <1%. WoLF PSORT was used to predicate subcellular localizations of identified proteins (Horton et al., 2007). AgriGOv2 were used for Gene Ontology enrichment analysis of proteins with differential Khib modification level (Tian et al., 2017). Metabolic pathways and biochemical signals transduction pathways were predicted by the Kyoto Encyclopedia of Genes and Genomes (KEGG) and KOBAS 3.0 (Kanehisa and Goto, 2000; Bu et al., 2021). PlantCyc enrichment analysis was performed by PlantGSAD (Ma et al., 2021). *p*-Value < 0.05 (Fisher’s exact test) was used as the threshold to determine the significant enrichments of GO and KEGG pathways.

### Chromatin immunoprecipitation and ChIP-qPCR

ChIP was performed as described previously with minor modification (Zhang et al., 2017). 10 g of maize stem was homogenized in liquid nitrogen and re-suspended by TBS. The nuclei were purified in sucrose gradient and digested by MNase (Sigma). The digested nucleosome samples were incubated with 4% protein A Sepharose (GE Healthcare Bio-Sciences) for 3 h and centrifuged. Pan anti-Khib (PTM Biolabs, PTM-801) and pan anti-H4ac (Millipore, 06-598) was incubated with supernatant. The sample was incubated with 25% protein A agarose gel for 2.5 h. After centrifugation, the precipitate is eluted with an elution buffer to wash the eluted immune complex. The immunoprecipitated DNA was extracted by phenol/chloroform extraction and ethanol precipitation. Quantitative real-time PCR analysis of ChIPed DNA was performed on the Bio-Rad touch real-time PCR detection system to determine the relative enrichment factor of the modified histone-related sequences in the binding part. UBQ1 gene was used as an internal reference. Primers were designed and listed in Supplementary Table S3.

### Immunoblotting analysis

Total proteins were extracted as described previously with minor modification according to Zhao et al. (2020). Then, proteins mixed with loading buffer were boiled for 5 min. Then, the proteins were separated by 12% SDS-PAGE and the separated proteins were transferred to polyvinylidene difluoride (PVDF) membrane (Millipore). The membranes were blocked (5% milk dissolved in 1× TBST) for 2 h at room temperature, and incubated at 4°C with anti-H3 (Abcam, ab1791), anti-H3K9ac (Abcam, ab10812), anti-H4K8hib (PTM Biolabs, PTM-805), pan anti-Khib (PTM Biolabs, PTM-801), pan anti-Kcr (PTM Biolabs, PTM-502), and pan anti-Ksucc (PTM Biolabs, PTM-419) antibodies overnight. The membrane was washed three times with TBST for 10 min each time, and then incubated with horseradish peroxidase-labeled secondary antibody at room temperature for 2 h. The membranes were washed three times with TBST, incubated in ECL for 1 min, and examined using automatic chemiluminescence image analysis systems.

### RNA extraction and quantitative real-time PCR

The same samples used in proteomics were frozen in liquid nitrogen for RNA extraction. The total RNA of the sample was separated by TRIZOL (Invitrogen) and purified by Qiagen RNA purification kit (Qiagen). Quantitative real-time PCR (qRT-PCR) used the cDNA reversely transcribed from the samples collected at 0 and 2 days after *F. graminearum* infection as the template. According to the manufacturer’s instructions, the TransStart Tip Green qPCR SuperMix kit was used for qRT-PCR experiment. UBQ1 gene was used as an internal reference to normalize by qRT-PCR. Then, comparative Ct analysis (2^–ΔΔCt^) of each gene was employed and its relative expression levels at different time points, and quantitative data were expressed as mean ± standard error of mean (SEM). Primers used in this study were designed and listed in Supplementary Table S3.

## Results

### Proteome-wide analyses revealed lysine 2-hydroxyisobutyrylome in maize

**Figure 1:**
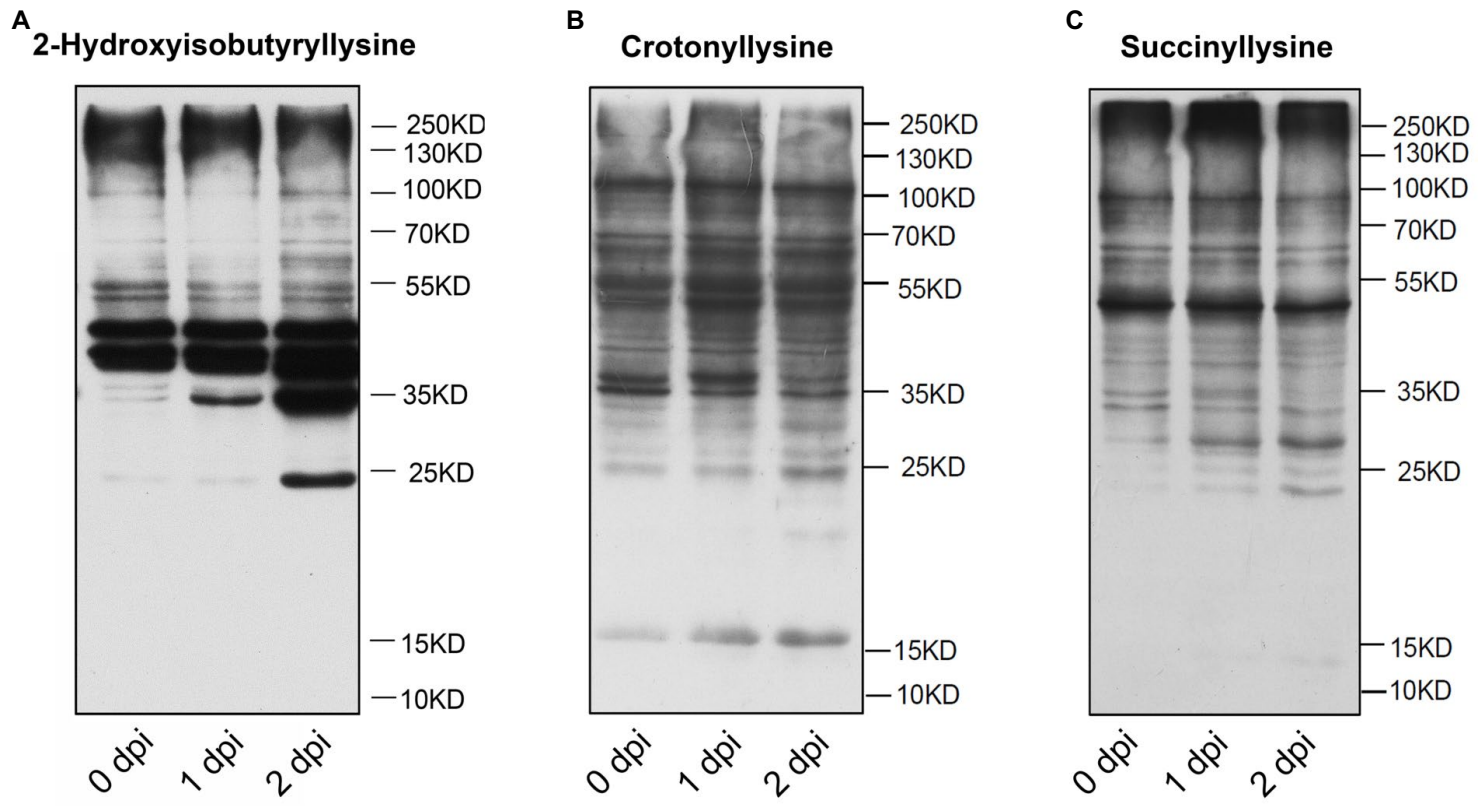
Immunoblotting analysis of total protein 2-hydroxyisobutyrylation (Khib) **(A)**, crotonyllysine (Kcr) **(B)**, succinyllysine (Ksucc) **(C)** levels during *Fusarium graminearum* infection in maize stems.

**Figure 2:**
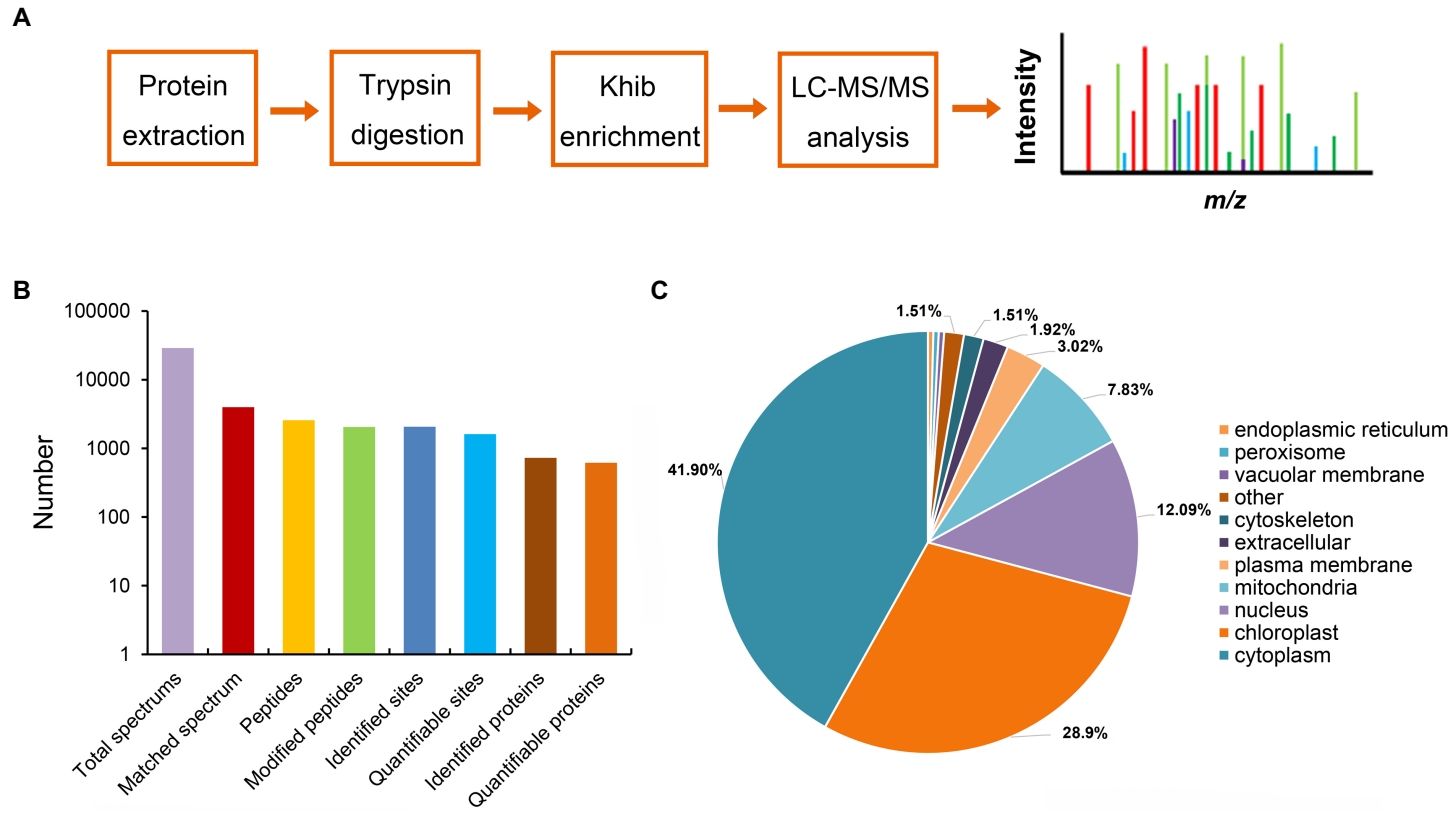
Proteome-wide identification and properties of Khib sites in maize. **(A)** Workflow of proteomics analysis. **(B)** Statistical analyses of the Khib sites, proteins, and peptides. **(C)** Subcellular localization of proteins with Khib modifications predicted by WoLF PSORT.

Post-translational modification (PTM) plays a key role in the regulation of protein function in maize, such as protein acetylation is involved in the biological process of responding to *Cochliobolus carbonum* infection (Waszczak et al., 2015; Walley et al., 2018). To investigate the distribution of post-translational modification in the proteins of maize, we performed western blot assaying using pan-Khib, pan-Kcr, and pan-Ksucc antibodies (Figure 1). The immunoblotting results showed multiple bands from the proteins in different infection stage, suggesting that Khib (Figure 1A; Supplementary Figure S1A), Kcr (Figure 1B; Supplementary Figure S1B), and Ksucc (Figure 1C; Supplementary Figure S1C) are widely distributed in maize stems. Compared with Kcr and Ksucc modifications, Khib modification was significantly increased after *F. graminearum* infection. To examine the Khib modification on histones, the blotting results displayed distinguished bands at 10–17 kD where the histones are located by increasing exposure time (Supplementary Figure S2). Therefore, proteomics-based Tandem Mass Tag (TMT) labeling technology with Khib peptides enrichment was conducted to explore the Khib modification sites in 0 and 2 days post inoculation (dpi) with *F. graminearum* in maize stems (Figure 2A). After a systematic analysis of Khib modification on proteins isolated from 0 and 2 dpi in maize stems, 2,066 Khib-modified peptides on 728 proteins were identified, and most of these proteins had 1–7 Khib modified sites (Figure 2B; Supplementary Figure S3A). Khib modified peptides are generally <19 amino acids (Supplementary Figure S3B). Peptide mass error and principal component analysis (PCA) analysis indicated that the proteomic data were reliable (Supplementary Figures S3C,D). To explore the subcellular localization of the proteins with Khib modification in maize, we predicted the localization of Khib modified proteins in different cell compartments by bioinformatic method. The results showed that 41.9% proteins located in cytoplasm, 28.9% in chloroplast, 12.09% in nucleus, and 7.83% in mitochondria, which suggested that these Khib-modified proteins might play an important role in cellular metabolic processes (Figure 2C). In addition, a small amount of proteins was also located in the plasma membrane (3.02%), extracellular (1.92%) and other locations, suggesting that 2-hydroxyisobutyryl modified proteins might play biological functions in different cellular components (Figure 2C).

### Identification of histone Khib modifications in maize

**Figure 3:**
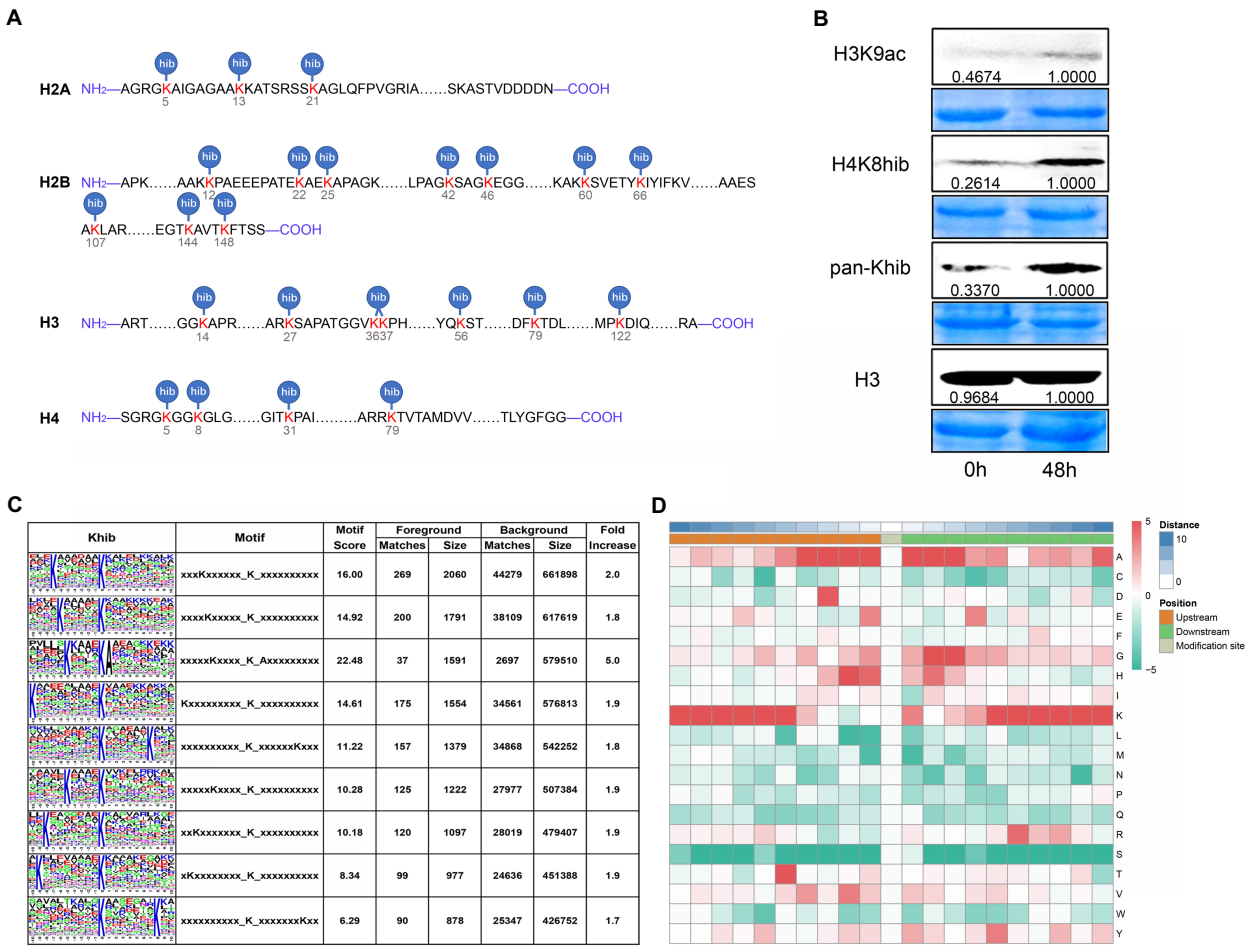
Identification of Khib modification sites on histones and Khib motifs. **(A)** Khib modification sites on histones identified by proteomics data. **(B)** Immunoblotting analysis of histone protein H3K9ac and Khib levels with histone deacetylase inhibitor (Trichostatin A, TSA) treatment. Histone H3 was used as a loading control. **(C)** Identification of 2-hydroxybutyrylation motifs by motif-x. The height of each letter corresponds to the frequency of that amino acid residue in that position. **(D)** Heatmap of the amino acid composition of the Khib modification sites, red and green, respectively, showed the enrichment and reduction of amino acids at each position (right letter) on both sides (from −10 to +10) of the Khib sites.

Lysine 2-hydroxyisobutyrylation (Khib) on histones is widespread in plants, such as *Arabidopsis*, rice, *Physcomitrella patens*, and wheat (Meng et al., 2017; Xue et al., 2020; Bo et al., 2021; Zhang et al., 2021). However, Khib modification on histones in maize had not been identified. Based on the proteomic data, we identified 24 Khib sites in the core histone throughout both the N termini, core regions of the histones, and C termini, and 10 of 24 Khib sites occurred on H2B (Figure 3A). Comparison with published data, the histone Khib sites in maize were highly conserved from mammal to plant, which suggested Khib modification was a widespread histone mark. However, we found for the first time that some histone Khib sites could be modified, such as H2AK5, H2BK12, H3K14, H4K5, H4K8, etc., which occurred in mammals (Figure 3A). In order to explore whether HDACs were the key enzymes to regulate Khib modification in maize, we analyzed the changes of Khib modification on histones in maize seedlings treated with histone deacetylase inhibitor (trichostatin A, TSA). We observed an obvious increase in H3K9ac, H4K8hib, and Khib band intensity after TSA treatment (Figure 3B). These results suggest that histone acetylation and 2-hydroxyisobutyrylation might have synergistic effect on gene expression.

### Motif analysis of the identified modified peptides in maize

In order to explore the conserve amino acid sequence around the 2-hydroxyisobutyrylation modification site, we analyzed the conserved motifs in the upstream and downstream 10 amino acid residues with lysine as the center by motif-x algorithm. Totally, we identified 9 conserved motifs around Khib modified sites in maize, included KxxxxxKhib, KxxxxxxKhib, KxxxxxxxxxKhib, and a non-polar amino acid preference motif KxxxxKhibA (x represented a random amino acid residue) (Figure 3C). Then, heatmap result of conserve amino acid enrichment analysis showed that lysine (K) residues enriched from positions −10 to −4 and from +3 to +10 around Khib modified sites, while glutamate (G) and histidine (H) at the −3 and +3 positions (Figure 3D). Contrary to serine (S), Alanine (A) appeared in almost all positions from −10 to +10 positions, excepted +6 position where R (arginine) occurred more frequently (Figure 3D).

### Functional enrichment analysis of Khib modified proteins during *Fusarium graminearum* infection in maize

**Figure 4:**
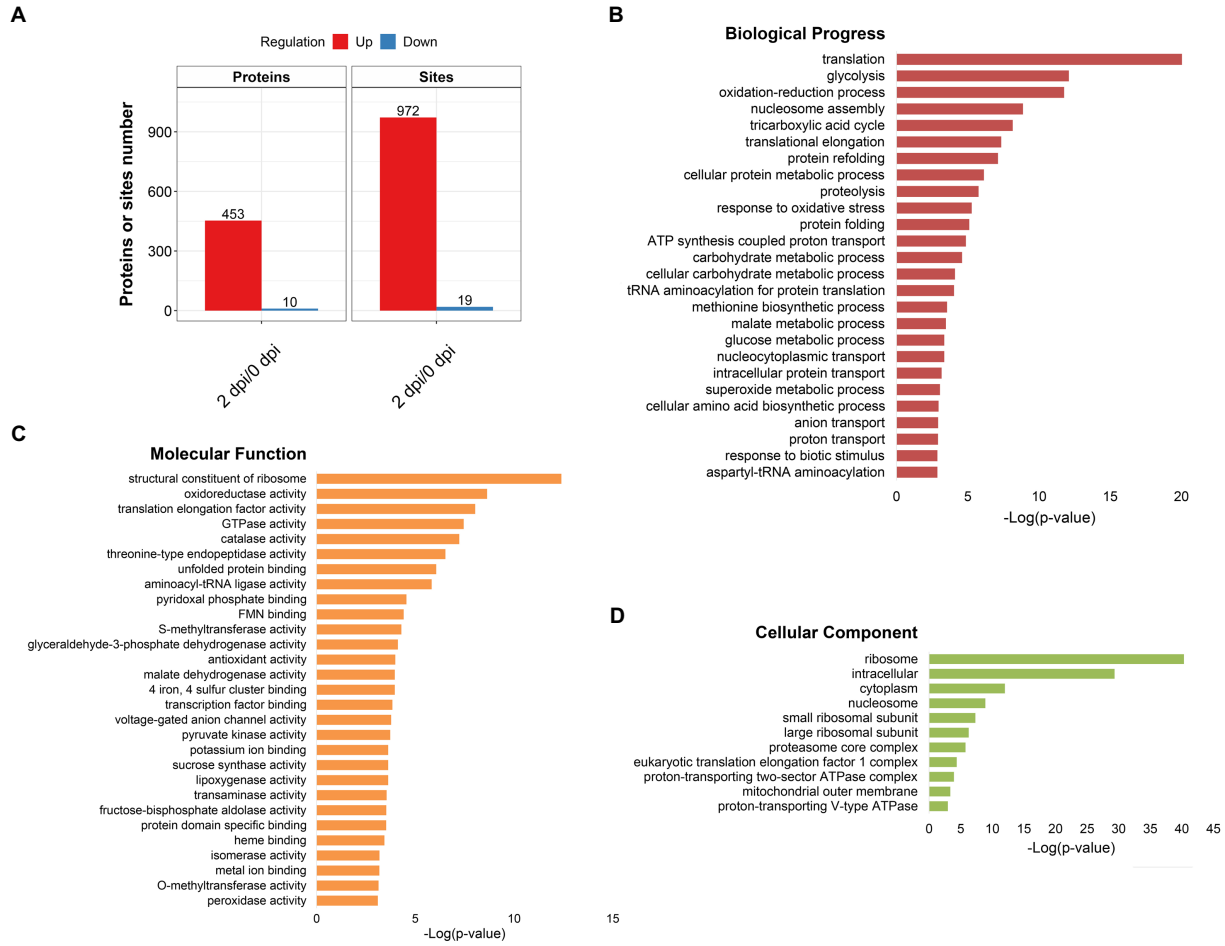
Identification and functional classification of Khib-modified proteins. **(A)** Numbers of up- and down-regulation proteins and Khib modification sites. **(B–D)** Gene ontology enrichment of upregulated differentially expressed proteins between 0 and 2 dpi. Biological progress **(B)**, Molecular function **(C)**, and Cellular component **(D)**. Fisher’s exact test, value of p was adjusted using the Benjamini-Yekutieli method, FDR < 0.05.

**Figure 5:**
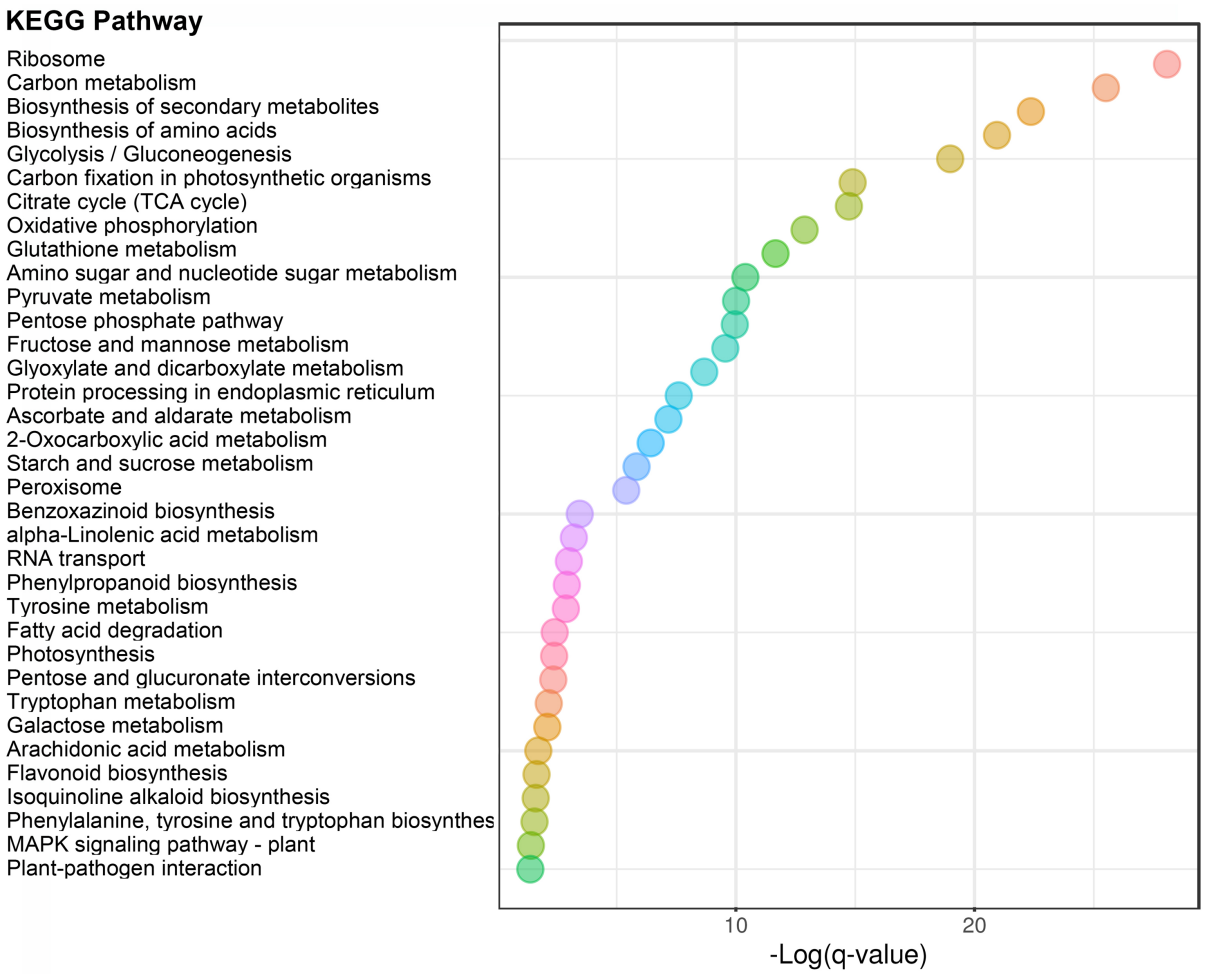
KEGG enrichment analysis of upregulated Khib-modified proteins between 0 and 2 dpi. The abscissa indicates the degree of significant enrichment.

**Figure 6:**
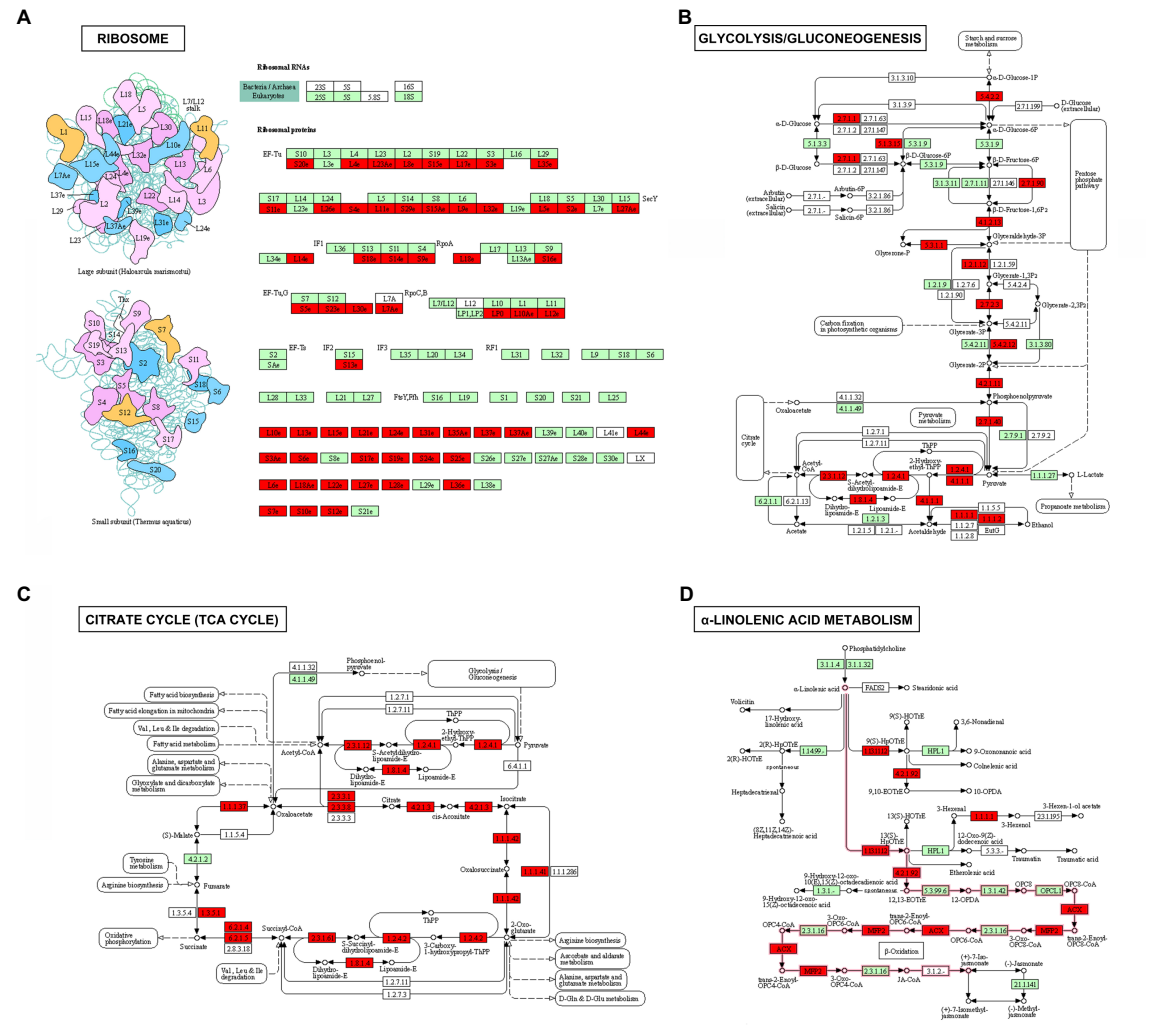
Upregulated Khib-modified proteins enriched in ribosome **(A)**, glycolysis/gluconeogenesis **(B)**, TCA cycle **(C)**, and alpha-linolenic acid metabolism **(D)** in maize. Green represents the enzyme or protein of maize in this KEGG pathway and the 2-hydroxyisobutyrylated enzymes or proteins identified are highlighted in red.

In order to analyze the function of 2-hydroxyisobutyrylation in the process of maize disease resistance, we used *F. graminearum* to infect maize stems, and statistically analyzed the differences of 2-hydroxyisobutyrylation modified proteins and modified sites on day 0 and day 2 after *F. graminearum* infection. The results showed that, the Khib modification levels of 453 proteins were upregulated, including 972 Khib modification sites (fold-change > 1.3, *p* < 0.05) (Figure 4A). Only a small number of proteins and Khib sites were down-regulated, including 10 proteins and 19 modification sites. The above results showed that a considerable number of Khib modification levels were changed after *F. graminearum* infection, suggesting that 2-hydroxyisobutyrylation might play an important role in maize resistance to *F. graminearum* infection. To further elucidate the role of 2-hydroxyisobutyrylation in maize resistance to *F. graminearum* infection, GO enrichment analysis of differentially modified Khib proteins (upregulated) was performed. The results showed that in the biological process, proteins with different 2-hydroxyisobutyrylation level were mainly involved in protein translation, protein folding, glycolysis, nucleosome assembly, TCA cycle, and response to biotic stress (Figure 4B). GO enrichment analysis based on molecular function showed that the different 2-hydroxyisobutyrylation proteins were closely related to the structural components of ribosomes, oxidoreductase activity, lipoxygenase activity, transport enzyme activity and transcription factors (Figure 4C). In the context of cellular component, these proteins were most enriched in ribosomes, followed by intracellular and cytoplasmic proteins (Figure 4D). Then, KEGG pathway enrichment analysis was performed with 453 upregulated Khib modified proteins from the respective proteomes to analyze the key enriched metabolic pathways in response to *F. graminearum* infection. The results showed that the Khib modified level upregulated proteins were mainly involved in multiple key metabolic processes, including ribosome, carbohydrate metabolism, glycolysis/gluconeogenesis, TCA cycle, oxidative phosphorylation, glutathione metabolism, amino sugar and nucleotide sugar metabolism, starch and sucrose metabolism (Figures 5, 6). In addition, these proteins with higher Khib modification level also enriched in metabolic pathways related to plant disease resistance, such as peroxisome, benzoxazinoid biosynthesis, phenylpropanoid biosynthesis, alpha-linolenic acid metabolism (jasmonic acid synthesis), phenylalanine, tyrosine and tryptophan biosynthesis, and plant-pathogen interaction (Figures 5, 6). We also performed enrichment analysis based on plant metabolic pathway databases (PlantCyc), which showed the similar results with KEGG (Supplementary Figure S4). Interestingly, alpha-linolenic acid metabolism (jasmonic acid synthesis pathway) were enriched in the upregulated Khib proteins, which suggested that Khib modification was involved in jasmonic acid synthesis, and further participated in plant disease resistance. All of the functional enrichment analysis indicated that 2-hydroxyisobutyrylation played an important role in maize resistance to *F. graminearum* infection.

### Regulation of pathogenesis-related gene expression by Khib modification

**Figure 7:**
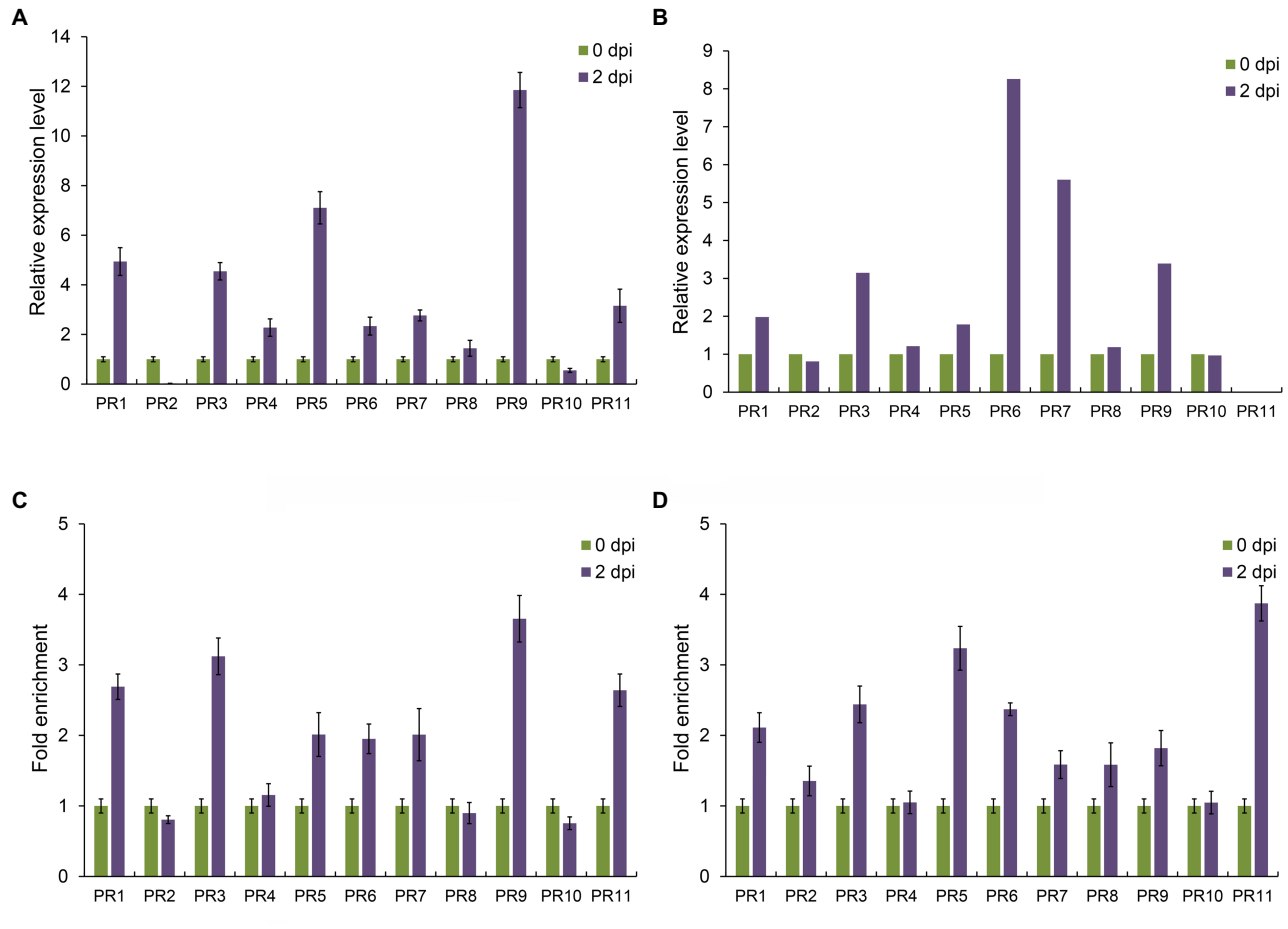
2-Hydroxyisobutyrylation affects gene expression of pathogenesis-related protein in maize. **(A)** Relative expression levels of PR genes under *Fusarium graminearum* infection. Data are the mean ± SEM of three independent experiments. **(B)** Relative expression levels of PR proteins under *F. graminearum* infection based on proteomics data. **(C)** Fold enrichment of histone Khib levels at the PR gene bodies after *F. graminearum* infection in maize. Data are the mean ± SEM of three independent experiments. **(D)** Fold enrichment of histone H4 acetylation levels at the PR gene bodies after *F. graminearum* infection in maize. Data are the mean ± SEM of three independent experiments.

Pathogenesis-related proteins (PRs) are a class of proteins that play important roles after pathogen infection in plants (Sels et al., 2008). Khib modification was involved in maize resistance to pathogen infection and could modify lysine on histones. Then, whether Khib modification also participate in regulating gene expression through modifying histone lysine? Therefore, we performed RNA extraction and chromatin immunoprecipitation (ChIP) experiments to detect PR genes expression and Khib modification status by quantitative real-time PCR systems. Based on the maize genome annotation, we identified 11 PR genes and found that most of them were upregulated after *F. graminearum* infection, except PR2 and PR10 (Figure 7A). Our proteomics data (PXD024342) also proved that most of these PR proteins participated the response to *F. graminearum* infection, while no PR11 signal was detected (Figure 7B; Bai et al., 2021). In *Arabidopsis*, Khib modification on histones is mainly enriched in the gene body region after the transcription start sites (Zheng et al., 2021). Thus, ChIP-qPCR primers used in this study were designed form +1 to +3 nucleosome positions behind transcription start sites. ChIP-qPCR results showed that Khib and Kac modification level of PR genes on histones were also upregulated after *F. graminearum* infection (Figures 7C,D), which suggested that histone Khib modification might been involved in the process of regulating gene expression and affecting plant disease resistance by coordinated with histone acetylation.

## Discussion

In recent years, with the continuous understanding of the significance of PTMs, more and more new forms of modification on lysine residues have been discovered and proved to be related to regulatory functions (Moellering and Cravatt, 2013), for example, lysine succinylation (Ksucc), butyrylation (Kbu), acylation (Kpr), crotonylation (Kcr), malonylation (Kma), β-hydroxybutyrylation (Kbhb) and 2-hydroxyisobutyrylation (Khib) (Lin et al., 2012; Huang et al., 2016; Lu et al., 2018). Like acetylation (Kac), these emerging post-translational modifications are classified as lysine acylation and have been shown to occur on multiple proteins involved in a variety of cellular metabolic processes (Balparda et al., 2021; Okada et al., 2021). Khib is a newly discovered post-translational modification of proteins, which plays a crucial role in the regulation of chromatin function, energy synthesis and metabolism and other biological processes (Dai et al., 2014; Huang et al., 2018; Dong et al., 2019). In plant, Khib modification was identified in *Arabidopsis*, rice, *Physcomitrella patens*, rhubarb, and wheat (Choudhary et al., 2014; Yu et al., 2017; Lin and Caroll, 2018; Muller, 2018; Zhao et al., 2018). However, there have been no such reports in maize. In this study, we first generated genome-wide maps of Khib modification in maize, and 2,066 Khib-modified peptides on 728 proteins were identified (Figure 2B), suggesting that Khib was a very conserved protein modification in eukaryotes. Previous studies have shown that proteins with Khib modification are found to be involved in many important biological processes, such as glycolysis, TCA cycle, protein synthesis, and ribosomal activity, and this is also the case in plants (Huang et al., 2017, 2018; Chen et al., 2021; Zhang et al., 2021). However, does Khib also play an important role in plant disease resistance? At present, studies in rice have shown that the Khib modification state of many proteins have been changed after *U. virens* infection, which indicates that Khib is involved in rice resistance to pathogen infection (Chen et al., 2021). In our study, the level of Khib modification increased with the infection time of *F. graminearum* in maize (Figure 1), while Khib modification level showed a reduction with *U. virens* infection in rice flowers (Chen et al., 2021). We thought that it may be caused by the type of pathogens, and the difference of infection time and tissues in maize. Functional analysis results of the proteins with higher Khib modification level were enriched not only in ribosome, glycolysis, TCA cycle, protein synthesis, but also in peroxisome, benzoxazinoid biosynthesis, phenylpropanoid biosynthesis, jasmonic acid synthesis, tyrosine and tryptophan biosynthesis, the secondary metabolic processes closely related to plant disease resistance (Figures 4, 5). In addition, Khib modification levels of 10 proteins were down-regulated after *F. graminearum* infection, which were involved in abscisic acid stress, pyridoxal phosphate transport, tiller repression and remorin family protein and suggested that these proteins might be important to the balance between growth and immune process. Transcriptomics and proteomics data proved that PR proteins were upregulated after *F. graminearum* infection (Figure 7). We also demonstrated that 2-hydroxyisobutyrylation on histones was closely related to PRs gene expression level, suggesting that Khib on histones was involved in plant disease resistance.

As a key epigenetic marker, histone modifications (methylation, acetylation, etc.) can maintain chromatin structure and be considered to be associated with gene expression in many important biological processes in plant, such as flower transformation, cell differentiation, gametogenesis and response to abiotic/biotic stresses (Liu et al., 2014; Deng et al., 2016). Moreover, the different modification types at different histone sites function in a variety of ways (He et al., 2011). Here, we identified 24 histone Khib sites and provide a genome-wide map of Khib modification on histones in maize. Compared with other plant species, the number of sites that undergo Khib modification on histone was different (Chen et al., 2021; Qi et al., 2021; Zhang et al., 2021; Zheng et al., 2021). However, we speculated that this phenomenon might be due to the interference of the input sample and the error in the process of mass spectrometry identification. In spite of this, we believed that Khib modification sites on histones were conserved and had great overlaps with acetylation. In this study, we first proved that histone 2-hydroxyisobutyrylation coordinated with acetylation could regulate PR genes expression levels (Figure 7). Histone deacetylase inhibitor (TSA) treatment experiments also supported this conclusion (Figure 3B). Is histone lysine acetylation and 2-hydroxyisobutyrylation involved in glycolysis, TCA cycle, starch biosynthesis, and other metabolic processes by regulating, thereby affecting maize disease resistance? Further experimental verification is needed.

Overall, 2-hydroxyisobutyrylation, as a new type of protein modification, participates in the process of maize resistance to pathogen infection, which was of great significance to the study of plant disease resistance.

## Data availability statement

The datasets presented in this study can be found in online repositories. The names of the repository/repositories and accession number(s) can be found at: https://www.ebi.ac.uk/pride/archive/, PXD030131.

## Author contributions

KZ, JX, and JD designed the experiments. KZ, HC, HS, and JZ analyzed the proteomic data. KZ, YM, HB, LY, XP, and FZ performed maize planting experiment and, ChIP-qPCR and western blotting assays. KZ, JX, and JD organized the results and wrote the paper. All authors contributed to the article and approved the submitted version.

## Funding

This work was supported by the National Natural Science Foundation of China (31901864), State Key Laboratory of North China Crop Improvement and Regulation (NCCIR2020ZZ-9), Natural Science Foundation of Hebei Province (C2019204141), Research Project of Science and Technology in Universities of Hebei Province (BJK2022006), China Agriculture Research System (CARS-02), Key Research and Development Projects of Hebei (19226503D), and Central Government Guides Local Science and Technology Development Projects (216Z6501G and 216Z6502G).

## Conflict of interest

The authors declare that the research was conducted in the absence of any commercial or financial relationships that could be construed as a potential conflict of interest.

JHD declared a shared affiliation with the authors at the time of the review.

## Publisher’s note

All claims expressed in this article are solely those of the authors and do not necessarily represent those of their affiliated organizations, or those of the publisher, the editors and the reviewers. Any product that may be evaluated in this article, or claim that may be made by its manufacturer, is not guaranteed or endorsed by the publisher.

## Supplementary material

Supplementary Figure S1 Coomassie brilliant blue staining results of total protein 2-hydroxyisobutyrylation (Khib) **(A)**, crotonyllysine (Kcr) **(B)**, succinyllysine (Ksucc) **(C)** levels during *F. graminearum* infection in maize stems.

Supplementary Figure S2 Western blotting analysis of 2-hydroxyisobutyrylation (Khib) on histones with *F. graminearum* infection in maize.

Supplementary Figure S3 QC validation of MS data. **(A)** Number of modification sites per protein. **(B)** Peptide length distribution. **(C)** Mass error distribution of all identified peptides. **(D)** Principal Component Analysis (PCA) of the total proteome data from maize stem at 0 and 2 days after *F. graminearum* infection. Protein samples at each time point have three replicates.

Supplementary Figure S4 Enrichment analysis of proteins with higher Khib modification levels in 2 dpi maize stem, based on plant metabolic pathway databases (PlantCyc).

Supplementary Table S1 MS/MS spectrum database search analysis of proteins and Khib modified sites identified during *F. graminearum* infection at 0 and 2 dpi in maize stem.

Supplementary Table S2 List of proteins with differentially modified Khib sites induced by *F. graminearum* infection at 0 and 2 dpi in maize stem.

Supplementary Table S3 Primers used for qRT-PCR and ChIP-qPCR in this study.
