# Supplementary figures and images for: Global analysis of lysine 2-hydroxyisobutyrylation during Fusarium graminearum infection in maize
Source: Front Plant Sci. 2022 Sep 15;13:1000039. doi: 10.3389/fpls.2022.1000039 (PMC9521605; doi:10.3389/fpls.2022.1000039)

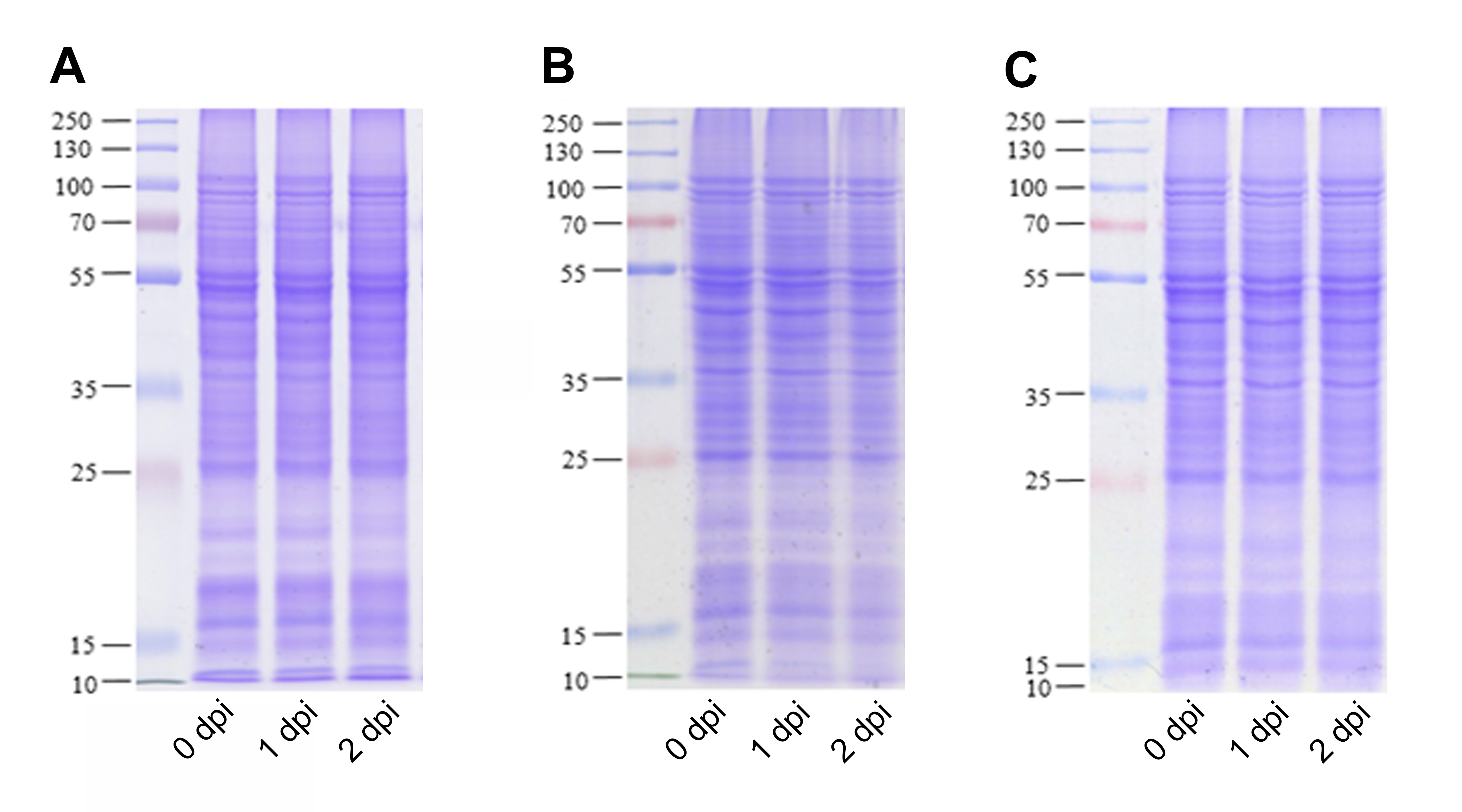

Supplement: Supplementary Figure S1 — Coomassie brilliant blue staining results of total protein 2-hydroxyisobutyrylation (Khib) (A), crotonyllysine (Kcr) (B), succinyllysine (Ksucc) (C) levels during F. graminearum infection in maize stems. [file Image_1.JPEG]

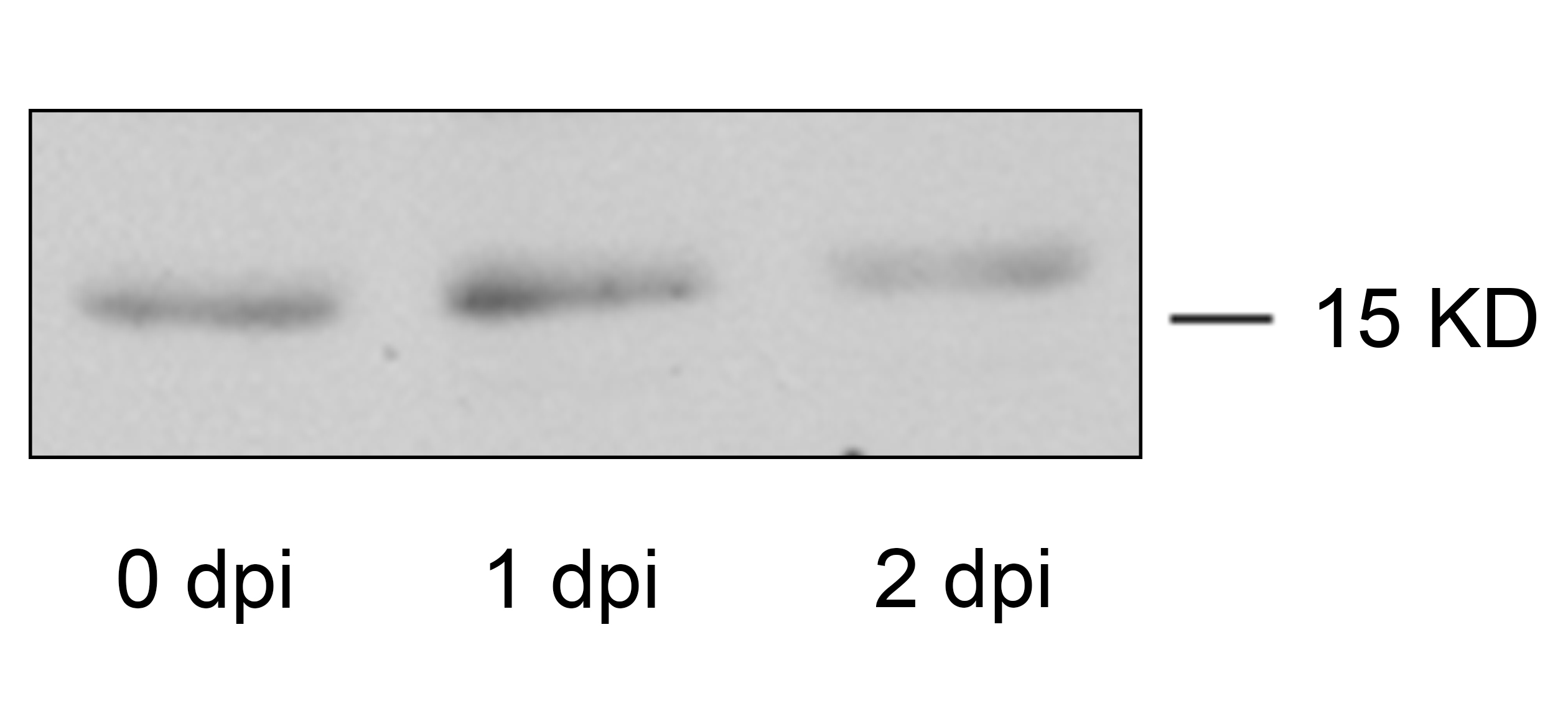

Supplement: Supplementary Figure S2 — Western blotting analysis of 2-hydroxyisobutyrylation (Khib) on histones with F. graminearum infection in maize. [file Image_2.JPEG]

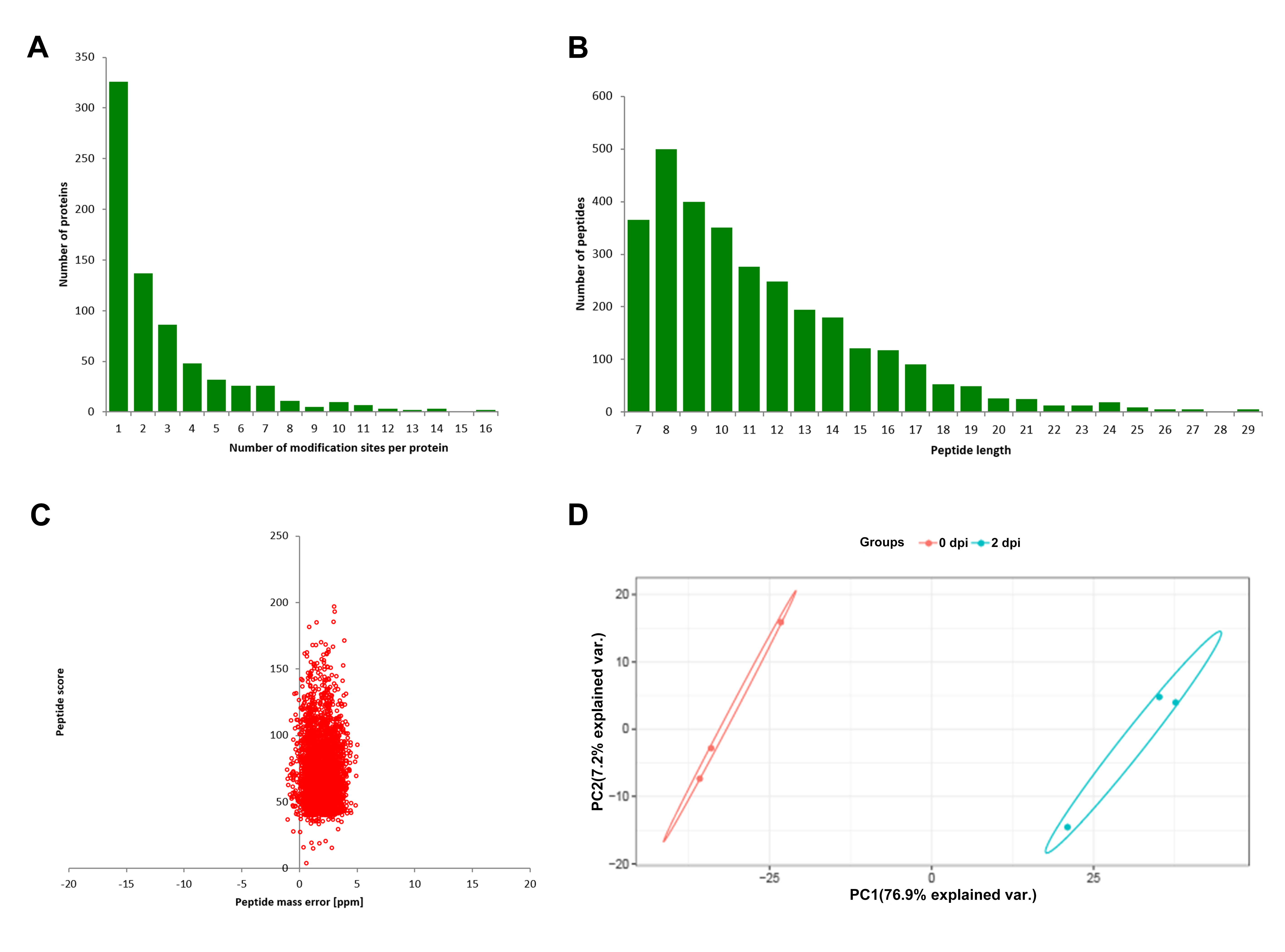

Supplement: Supplementary Figure S3 — QC validation of MS data. (A) Number of modification sites per protein. (B) Peptide length distribution. (C) Mass error distribution of all identified peptides. (D) Principal Component Analysis (PCA) of the total proteome data from maize stem at 0 and 2 days after F. graminearum infection. Protein samples at each time point have three replicates. [file Image_3.JPEG]

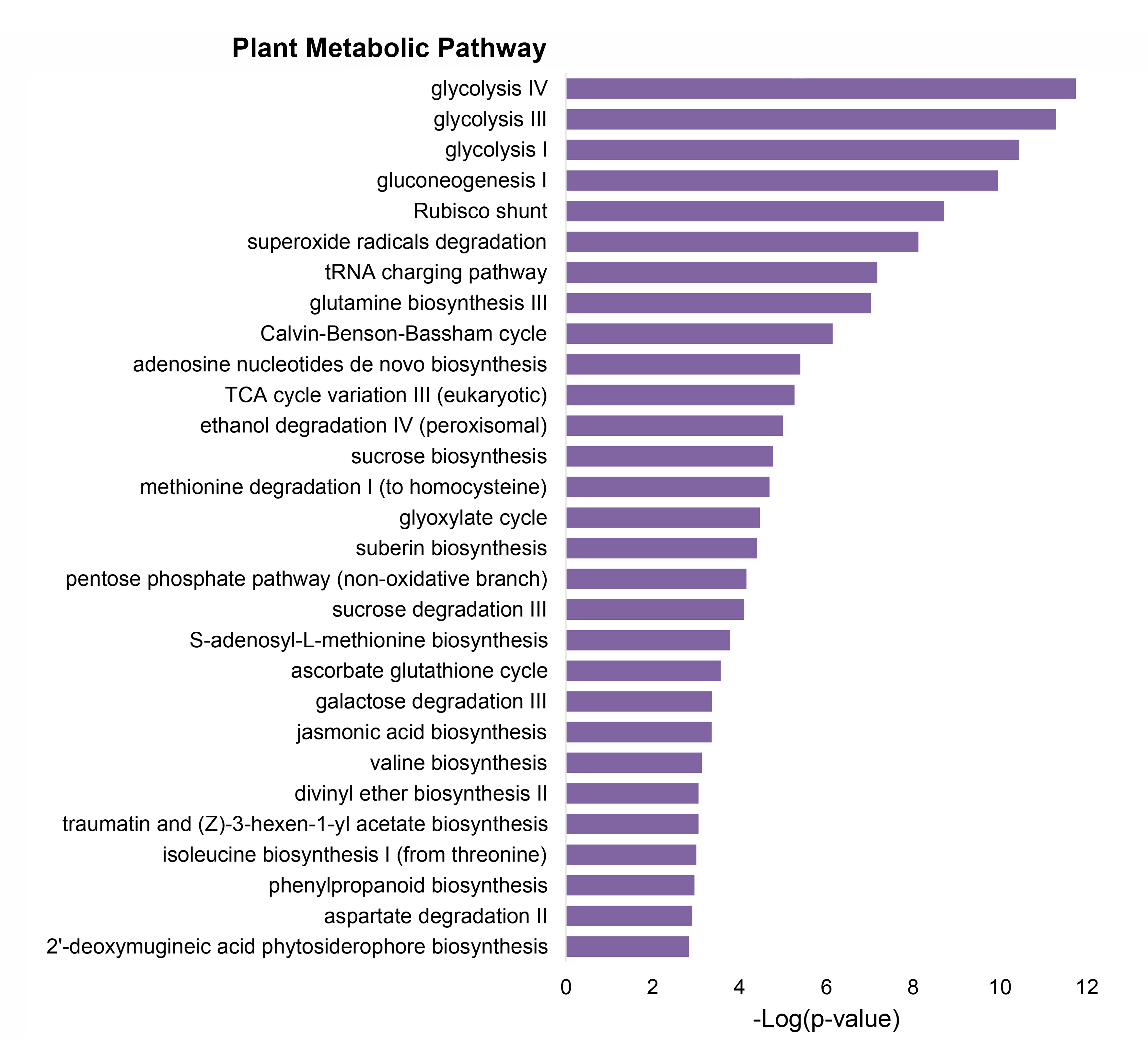

Supplement: Supplementary Figure S4 — Enrichment analysis of proteins with higher Khib modification levels in 2 dpi maize stem, based on plant metabolic pathway databases (PlantCyc). [file Image_4.JPEG]
